# Tracing the evolution of amniote chromosomes

**DOI:** 10.1007/s00412-014-0456-y

**Published:** 2014-03-25

**Authors:** Janine E. Deakin, Tariq Ezaz

**Affiliations:** Institute for Applied Ecology, University of Canberra, Canberra, 2601 Australia

**Keywords:** Comparative genomics, Chromosome painting, Ancestral karyotype, Reptile, Bird, Mammal

## Abstract

A great deal of diversity in chromosome number and arrangement is observed across the amniote phylogeny. Understanding how this diversity is generated is important for determining the role of chromosomal rearrangements in generating phenotypic variation and speciation. Gaining this understanding is achieved by reconstructing the ancestral genome arrangement based on comparisons of genome organization of extant species. Ancestral karyotypes for several amniote lineages have been reconstructed, mainly from cross-species chromosome painting data. The availability of anchored whole genome sequences for amniote species has increased the evolutionary depth and confidence of ancestral reconstructions from those made solely from chromosome painting data. Nonetheless, there are still several key lineages where the appropriate data required for ancestral reconstructions is lacking. This review highlights the progress that has been made towards understanding the chromosomal changes that have occurred during amniote evolution and the reconstruction of ancestral karyotypes.

## Introduction

Chromosomes, the basic units into which DNA is packaged in a nucleus, have undergone changes in gene content and organization throughout evolution. The great diversity of chromosome numbers between different amniote species and even the contrasting division of macro and microchromosomes in most birds and non-avian reptiles presents an opportunity to study chromosome evolution to determine the timing and types of events that shaped the chromosomes of extant amniote species. This involves comparing chromosomes of different species to reconstruct the most likely chromosome arrangement in a common ancestor. Tracing such events can provided great insight into the evolutionary process and even the role chromosomal rearrangements play in phenotypic evolution and speciation.

Reconstruction of ancestral karyotypes at various positions along the amniote (reptiles, birds and mammals) phylogenetic tree has been made possible by the large number of cross-species chromosome painting and gene mapping studies that have been carried out over the last 20 years, and more recently from the availability of sequenced and anchored genomes. Reconstructions based on cross-species chromosome painting data provide the most basic ancestral plan, only permitting the arrangement of relatively large-scale evolutionary events to be traced. The limits of detection of chromosome painting also govern the evolutionary depth to which the reconstruction can be applied. For instance, chromosome paints generated from eutherian species (e.g. humans) fail to detect homology with marsupial or monotreme chromosomes (Graphodatsky et al. 2012) and therefore, ancestral karyotype reconstructions based solely on eutherian chromosome painting data are restricted to the eutherian lineage. Interestingly, a greater evolutionary depth is possible in the reptilian lineage, with chromosome probes derived from chicken able to detect homology to crocodile, turtle and lizard species (Kasai et al. 2012; Pokorna et al. 2012; Pokorna et al. 2011), which last shared a common ancestor over 200 million years ago (MYA). Combining chromosome painting data with gene mapping or whole genome data permits reconstructions over greater evolutionary time, enabling the evolutionary events that have occurred across amniotes to be determined. However, at this stage, there are only a limited number of species for which the appropriate detailed data is available. In addition, the species that have been mapped or sequenced are not necessarily the best representative species for a particular lineage, which could complicate the reconstruction process. Basically, there is no single approach that is able to provide all the answers but it is a matter of aptly using the available data.

Here, we review the advancements that have been made in this field using molecular cytogenetics and comparative genomics analysis, highlighting the data missing from key species and suggest approaches that can be taken to rapidly bridge these knowledge gaps.

## Amniote chromosome numbers

**Fig. 1:**
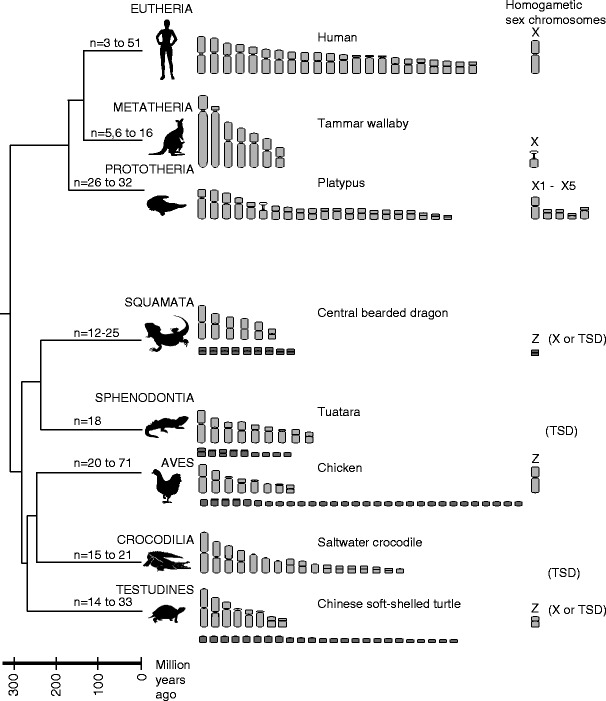
Amniote phylogeny showing haploid karyotypes for representative species. The range of haploid chromosomes numbers for each lineage are indicated on the branches (Christidis 1990; Hayman 1990; O’Brien et al. 2006; Olmo and Signorino 2005; Valenzuela and Adams 2011). Microchromosomes are indicated in *dark grey*. The sex chromosomes present in the homogametic sex are shown for representative species and alternatives present in each lineage are indicated. *TSD* temperature sex determination

Amniotes, which include birds, non-avian reptiles (herein referred to as reptiles) and mammals, last shared a common ancestor approximately 310 MYA. The chromosomes of each of these three major amniote lineages are strikingly different (Fig. 1), suggesting that their genomes have been subject to considerable rearrangement since last sharing a common ancestor.

Karyotypes of birds and most reptiles consist of up to ten pairs of macrochromosomes and a varying number of microchromosomes (Table 1). Birds have a particularly large number of microchromosomes. Reptiles are considered to be a karyologically heterogeneous group displaying high diversity in chromosome numbers and morphologies between and among groups (Olmo 2008). Among reptiles, crocodilians (Cohen and Gans 1970; Olmo 2008) and turtles (Olmo 2008; Olmo and Signorino 2005; Valenzuela and Adams 2011) have the most conserved karyotypes. In contrast, squamates reptiles (snakes and lizards, including legless lizards) have a high level of karyotypic variability in both morphologies and numbers observed. Snakes have relatively conserved chromosome numbers, which typically include eight pairs of macrochromosomes and ten pairs of microchromosomes. However, karyotypes have undergone frequent rearrangements including fission, fusion and repeat accumulation (Mengden and Stock 1980; O’Meally et al. 2010). Lizards (including legless lizards) seem to show the highest karyotypic variability among reptiles.

**Table 1:** The range of diploid numbers in amniotes (Christidis 1990; Hayman 1990; O’Brien et al. 2006; Olmo and Signorino 2005; Valenzuela and Adams 2011)

	Diploid chromosome numbers			Microchromosomes
	Smallest	Largest	Common^a^	
Birds	40	136–142	80	Yes
Crocodiles	30	42		No
Turtles	28	66		Yes
Snakes	30	42	36	Yes
Lizards	24	46		Yes^b^
Legless Lizards	30	50		Yes
Monotremes	52	64		No
Marsupials	10	32	14 and 22	No
Eutherians	6	102		No

^a^Only given for groups were there is a common diploid number

^b^Absent from some species

Mammalian karyotypes vary greatly between the prototherians (monotremes), metatherians (marsupials) and eutherians (placentals). Monotremes have karyotypes with high diploid numbers (Table 1). At first glance, it may appear that monotreme karyotypes are similar to those of reptilian species with eight large and many small chromosomes (Matthey 1949). However, the chromosome size range is much more continuous than that typically observed in reptiles and birds, and the small chromosomes are much larger than tiny microchromosomes (Van Brink 1959). In contrast, marsupial karyotypes mostly consist of several large chromosomes. Eutherian mammals show the most karyotypic diversity (Table 1) (O’Brien et al. 2006) and particular eutherians lineages, such as the rodents, gibbons, and canines have experienced extensive chromosome rearrangement.

Tracing the evolutionary history of amniote chromosomes will help us to understand the events that have led to the extraordinary diversity in karyotype morphology and chromosome number observed. This involves establishing the chromosome homology between representative species and reconstructing key ancestral karyotypes.

## The ancestral avian karyotype

**Fig. 2:**
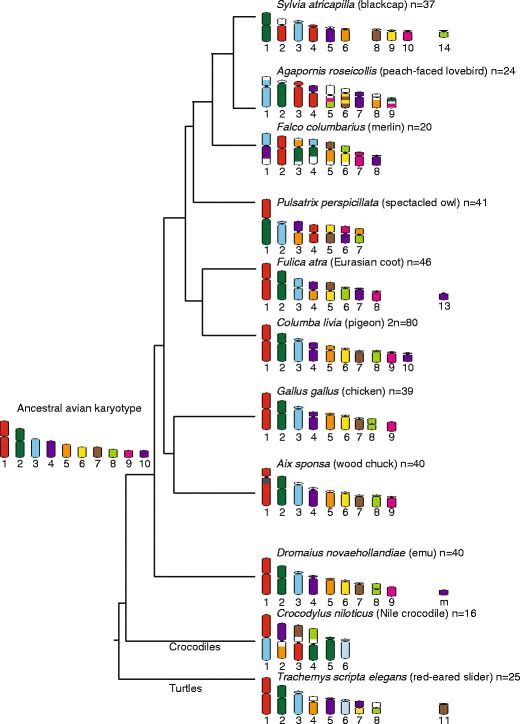
Homology identified in representative species across different avian lineages and outgroups (crocodile and turtle) using cross-species chromosome painting with probes derived from chicken chromosomes 1 to 9 (*depicted by different colours*). The predicted ancestral avian karyotype is indicated (Griffin et al. 2007). Chromosome homology was sourced from: *T .s. elegans* and *C. niloticus* (Kasai et al. 2012); *D. novaehollandiae* (Shetty et al. 1999); *A. sponsa*, *F. atra* and *S. atricapilla* (Nanda et al. 2011); *C. livia* (Derjusheva et al. 2004); *P. perspicillata* (de Oliveira et al. 2008); *F. columbarius* (Nishida et al. 2008); *A. roseicollis* (Nanda et al. 2007). *m* microchromosome

Avian karyotypes are divided into macro- and microchromosomes, with the macrochromosomes being relatively gene poor compared to the gene-rich microchromosomes (Burt 2002). In chicken (*Gallus gallus*), the most well-studied avian genome, the macrochromosomes make up approximately 70 % of the genome (Kasai et al. 2012). Chromosome homology between different avian orders has revealed a striking level of conservation of macrochromosomes between divergent taxa (Griffin et al. 2007). Cross-species chromosome painting, typically using probes generated from chicken chromosomes 1 to 9 and often the large Z chromosome, have shown that, in many cases, whole chromosomes have remained intact (de Oliveira et al. 2008; Derjusheva et al. 2004; Nanda et al. 2011; Nishida et al. 2008). This remarkable conservation is not restricted to the avian lineage but is even observed in outgroup species such as crocodiles and turtles (Kasai et al. 2012), which last shared a common ancestor with birds ∼230 MYA. The homology between chicken chromosomes and those of representatives of various avian orders is shown in Fig. 2. The high level of conservation has made it relatively easy to predict the ancestral avian karyotype (Griffin et al. 2007), even despite the uncertainty surrounding avian phylogeny and their relationship to turtles and crocodiles (Kasai et al. 2012).

Despite the infrequent occurrence of interchromosomal rearrangements detected by chromosome painting, comparisons of gene order from gene maps or sequenced and anchored genomes has demonstrated that there are intrachromosomal rearrangements (Skinner and Griffin 2012; Volker et al. 2010). This is exemplified by the comparison of the genome assemblies for chicken, zebra finch (*Taeniopygia guttata*) and turkey (*Meleagris gallopavo*), where inversions are evident and particular regions of the genome appear to be prone to rearrangement (Skinner and Griffin 2012). The Z chromosome is one chromosome that has been particularly susceptible to intrachromosomal rearrangements (Griffin et al. 2007). It is unclear why bird genomes are more prone to intrachromosomal than interchromosomal rearrangement but Skinner and Griffin (2012) propose that there may be an advantage for birds maintaining the association of certain syntenic blocks in interphase nuclei. Nonetheless, intrachromosomal rearrangements are capable of bringing about phenotypic change and therefore, may play an important role in speciation in the avian lineage. For example, an inversion on chromosome 2 in the white-throated sparrow (*Zonotrichia albicollis*) is responsible for behavioural differences in the population, providing an example of phenotypic variation generated by an intrachromosomal rearrangement and may even be an example of speciation in action (Thomas et al. 2008). Avian genome evolution has been thoroughly reviewed in recent years and we refer the reader to these reviews for further details (Ellegren 2010; Griffin et al. 2007).

### Avian microchromosomes

Evolution of bird macrochromosomes is now relatively well understood. However, our understanding of microchromosome evolution is lagging. Microchromosomes are thought to have evolved ∼400 million years ago, taking their ancestry to the common ancestors of amphibians and amniotes (Burt 2002). Gaining an in-depth understanding of the evolution of avian microchromosomes has been hampered by the difficulty in obtaining comparative data for these tiny chromosomes. Cross-species chromosome painting with chicken probes that has been so successful in identifying the homology of avian macrochromosomes has been problematic for microchromosomes, with it being difficult to obtain probes specific to individual microchromosomes by flow sorting (Griffin et al. 1999). Microdissection of individual chicken microchromosomes has produced probes for cross-species painting (Griffin et al. 1999; Grutzner et al. 2001) but these probes are unable to detect homology between distantly related species (Grutzner et al. 2001).

The limited number of studies using chicken microchromosome paints on other bird species has revealed that, in most cases, single microchromosomes in chicken are homologous to single microchromosomes in other species or probes derived from pools of chicken microchromosomes hybridise to the same number of microchromosomes in other species, such as goose (Griffin et al. 1999), pheasant (*Phasanius colchicus*) (Grutzner et al. 2001), turkey (Griffin et al. 2008), pigeon (*Columba livia*), chaffinch (*Fringilla coelebs*) and redwing (*Turdus iliacus*) (Derjusheva et al. 2004). This implies that synteny is conserved for microchromosomes, which is supported by mapping of chicken bacterial artificial chromosome (BAC) clones to duck (*Anas platyrhynchos*) (Fillon et al. 2007; Skinner et al. 2009) and quail (*Coturnix japonica*) (Kayang et al. 2006) microchromosomes, where pairs of BACs marking opposite ends of chicken microchromosomes mapped to single microchromosomes in the duck or quail.

Exceptions to this level of conservation can be found in species with atypical avian karyotypes, such as the stone curlew (*Burhinus oedicnemus*) (Nie et al. 2009), a member of a phylogenetically young group of birds with a low diploid number of 2*n* = 42 or the Harpy eagle (*Harpia harpyja*) with a 2*n* = 58 karyotype (de Oliveira et al. 2005). Stone curlew medium-size chromosomes share homology mostly with chicken microchromosomes, indicating that fusions contributed significantly to shaping the curlew karyotype (Nie et al. 2009). This is also supported by the hybridization of stone curlew chromosome paints onto chromosomes from species representing five different avian orders (Hansmann et al. 2009). In the Harpy eagle, Japanese mountain hawk-eagle (*Nisaetus nipalensis orientalis*) and three falcon species (*Falco tinnunculus*, *Falco peregrinus*, *Falco columbarius*), chromosome painting revealed tandem fusions between microchromosomes, as well as between microchromosomes and macrochromosomes (de Oliveira et al. 2005; Nishida et al. 2013; Nishida et al. 2008). This is also likely to be the case in the parrot *Agapornis roseicollis*, where there is some evidence of this from mapping of a chicken microchromosome probe to a macrochromosome (Nanda et al. 2007).

These interesting tiny, gene-rich chromosomes need to be the focus of future studies in order to more fully understand the evolution of avian genomes. A comparison of chicken and turkey intronic and cDNA sequence has indicated that microchromosomes have a higher rate of sequence evolution than the larger chromosomes, most likely influenced by the higher incidence of CpG sites on microchromosomes (Axelsson et al. 2005). It would be interesting to expand this type of analysis to many more species, particularly to species where fusions of ancestral microchromosomes have occurred. In order to do this, it is essential to have sequence anchored to chromosomes. Of the 11 bird genomes published to date, only a third of these have been at least partially anchored to chromosomes (Table 2). Although the growing list of bird genomes being sequenced using next generation technology will be valuable resources, their usefulness for understanding avian and amniote genome evolution will be limited without the input of additional information (Lewin et al. 2009).

**Table 2:** Published bird genome assemblies

Species	Common name	Percent anchored^a^	Reference
*Gallus gallus*	Chicken (red jungle fowl)	89	International Chicken Genome Sequencing C 2004
*Taeniopygia guttata*	Zebra finch	83^b^	Warren et al. 2010
*Meleagris gallopavo*	Wild turkey	83	Dalloul et al. 2010
*Ficedula albicollis*	Collared flycatcher	73	Ellegren et al. 2012
*Anas platyrhynchos domestica*	Pekin duck	26	Huang et al. 2013
*Amazona vittata*	Puerto Rican parrot	–	Oleksyk et al. 2012
*Ara macao*	Scarlet macaw	–	Seabury et al. 2013
*Columba livia*	Domestic rock pigeon	–	Shapiro et al. 2013
*Falco cherrug*	Saker falcon	–	Zhan et al. 2013
*Falco peregrinus*	Peregrine falcon	–	Zhan et al. 2013
*Geospiza magnirostris*	Large ground finch	–	Rands et al. 2013

^a^Percentage of genome assembly anchored to chromosomes

^b^Anchored to chromosomes or linkage groups

### Sex chromosome evolution

All birds have a ZZ male/ZW female sex chromosome system, with the Z chromosome conserved across Aves, from the Palaeognathae (Struthioniformes and Tinamiformes), such as the emu (Nishida-Umehara et al. 2007; Shetty et al. 1999), to the Neognathae, including those with more atypical avian karyotypes, such as falcons (Nishida et al. 2008). This indicates that avian sex chromosomes arose prior to the divergence of the Palaeognathae and Neognathae lineages. The W chromosome differs in size between different avian species, being almost identical to the Z in size in Palaeognathae, to being smaller and heterochromatic in many Neognathae (reviewed in Stiglec et al. 2007). In contrast to the more highly differentiated ZW pairs of Neognathae species, Palaeognathae Z and W chromosomes recombine over a large portion of the chromosome (Pigozzi and Solari 1997). However, the apparent lack of differentiation over the last 100 million years is not solely due to this recombination but may be a consequence of the absence of a dosage compensation mechanism (required to equalize Z-gene expression between males and females) in these species. In fact, the lack of such a mechanism may have constrained sex chromosome differentiation so that two copies of most genes, particularly those that are dosage sensitive, are maintained on the W chromosome (Adolfsson and Ellegren 2013).

Sequencing information of the Z and W chromosomes of different avian species will provide a more in-depth understanding of the evolution of these chromosomes. However, the challenge is in obtaining the highly repetitive W sequence. The assembly of chicken W in the published chicken genome covers only a tiny fraction (0.5 %) of the entire chromosome (International Chicken Genome Sequencing C 2004). About 7 % of the ZW genome has not been anchored to a chromosome and therefore is a source for potential W-specific sequence. By sequencing a male genome using the Illumina sequencing platform and aligning reads to the assembled ZW genome, more W-specific sequences have been identified (Chen et al. 2012). Hopefully, similar bioinformatic approaches and the continued development of sequencing technology capable of sequencing through repetitive regions will lead to a thorough understanding of bird sex chromosome evolution.

## Towards understanding the evolution of reptile genomes

Reptiles occupy a key position in vertebrate phylogeny by sharing the common ancestor to birds and mammals (Fig. 1). Therefore, they are likely to play a critical role by providing fundamental and basic information to better understand genome organization and evolution in birds and mammals. The first step in determining how the genomes of this karyotypically diverse group have evolved is to establish the level of conservation between different reptilian species, as well as more broadly with birds and mammals. Progress in this area has been slow compared to birds and mammals, but is rapidly gaining pace in this post-genomics era.

In comparison to birds and mammals, only a limited number of cross-species chromosome painting studies have been carried out to detect the level of conservation among particular groups of reptiles (Giovannotti et al. 2009; Pokorna et al. 2012; Trifonov et al. 2011). Nonetheless, these studies also discovered a gross level of conservation between avian and squamate genomes, suggesting retention of large homologous synteny blocks (HSBs) throughout the evolutionary history of reptiles (Pokorna et al. 2012). Cross-species chromosome painting has also been used to decipher inter-specific genome evolution in skinks (Scincidae), which detected strong conservation of chromosomes between five species with variable chromosome numbers, suggesting a monophyletic origin of the family Scincidae (Giovannotti et al. 2009). Similarly, cross-species chromosome painting in seven species of geckos, not only detected highly conserved karyotypes but also detected species specific rearrangements in the common house gecko (*Hemidactylus frenatus*) (Trifonov et al. 2011).

### Reptiles in the era of comparative genomics

Compared to other vertebrates, reptile genomes have had a late start in the area of comparative genomics but are rapidly catching up. Although whole genome sequences are available (Table 3) or in progress for an increasing list of reptilian species, none of these genomes, except that of the green anole (*Anolis carolinensis*), have been anchored to chromosomes, and even then only about 60 % of the sequence has been anchored (Alfoldi et al. 2011). Conversely, the genomes of most reptiles for which there are molecular cytogenetic maps (Table 4) are yet to undergo whole genome sequencing. These chromosome maps have provided significant insight into genome organization and evolution in amniotes, identifying an unprecedented level of conservation of amniote genomes. For example, gene mapping data in six reptiles revealed a high level of karyotypic conservation between birds and reptiles, implying retention, to a large degree, of the ancestral karyotype (Uno et al. 2012).

**Table 3:** Published reptile genomes

Species	Common name	Percent anchored^a^	Reference
*Anolis carolinensis*	Green anole lizard	60	Alfoldi et al. 2011
*Pelodiscus sinensis*	Soft-shelled turtle	–	Wang et al. 2013
*Chelonia mydas*	Green sea turtle	–	Wang et al. 2013
*Chrysemyspicta bellii*	Western painted turtle	–	Shaffer et al. 2013
*Python molurus bivittatus*	Burmese python	–	Castoe et al. 2013
*Ophiophagus hannah*	King cobra	–	Vonk et al. 2013

^a^Percentage of genome assembly anchored to chromosomes

**Table 4:** Reptile species with molecular cytogenetic maps

Species	Common name	Marker type	No. of markers	Reference
*A. carolinensis*	Green anole lizard	BAC	356	Alfoldi et al. 2011
*Elpaphe quadrivirgata*	Japanese four-striped rat snake	cDNA	183	Matsubara et al. 2012; Matsubara et al. 2006; Matsuda et al. 2005
*Pelodiscus sinensis*	Chinese soft-shelled turtle	cDNA	162	Uno et al. 2012; Matsuda et al. 2005
*Crocodylus siamensis*	Siamese crocodile	cDNA	131	Uno et al. 2012
*Pogona vitticeps*	Central bearded dragon	BAC	87	Young et al. 2013
*Varanus salvator macromaculatusi*	Water monitor lizard	cDNA	86	Srikulnath et al. 2013
*Leiolepis reevesii rubritaeniata*	Butterfly lizard	cDNA	54	Srikulnath et al. 2009
*Sphenodon punctatus*	Tuatara	BAC	21	O’Meally et al. 2009
*Varanus exanthematicus*	Savannah monitor lizard	cDNA	17	Srikulnath et al. 2013

Recently, Young et al. (2013) developed a cytogenetic map in an agamid lizard, the central bearded dragon (*Pogona vitticeps*) where every single chromosome, including the microchromosomes was anchored with at least one BAC clone/gene. This BAC anchored cytogenetic map provided a unique opportunity to perform high-resolution comparative genomic studies with the capacity to identify fine scale rearrangements. For example, this study revealed an intrachromosomal rearrangement on the long arm of chromosome 1 of *P. vitticeps* 1q and identified regions orthologous to the Chinese soft-shelled turtle, *Pelodiscus sinensis* ZW and lizard *A. carolinensis* XY sex chromosomes (Young et al. 2013).

### Reptile microchromosomes

One of the characteristic features of most reptilian karyotypes is the presence of a variable number of microchromosomes. Compared to avian microchromosomes, only little is known about the genomics of those from reptile species. Classical staining studies in many reptiles provided evidence of the presence of GC-rich sequences on microchromsomes, which are therefore likely to be gene-rich like their avian counterparts. However, staining studies in tuatara showed evidence of AT-rich sequences on microchromosomes (O’Meally et al. 2009). Hence, microchromosomes may have evolved independently multiple times in different lineages.

Analysis of reptilian microchromosomes at the sequence level, and subsequent comparisons with avian microchromosomes, thus presents an excellent opportunity to discover novel insight into their evolution. This has been performed for the green anole, where sequence has been assigned to six microchromosomes, and displays conserved synteny exclusively with chicken microchromosomes. Unlike the higher GC content of avian microchromosomes, there was no GC content difference between macro- and microchromosomes in the green anole (Alfoldi et al. 2011). Partial sequence, gene, and repeat content has also been determined for BAC clones mapping to the W chromosome (a microchromosome) and one mapping to an autosomal microchromosome in *P. vitticeps* (Ezaz et al. 2013). Such analysis incorporating more species is being made easier due to the advancement of cytogenetic techniques (such as manual microdissection), genome amplification and subsequent sequencing (e.g. Illumina) to generate quality sequence information for comparative studies. Sequencing chromosomes from DNA amplified from a single microchromosome has been found to produce higher quality sequence data compared to that from flow sorted microchromosomes (Ezaz, unpublished). This combined approach of microdissection and sequencing will identify gross homologies of microchromosomes between species and groups, as well as sequence composition, will help us to discover the occurrence of fine scale rearrangements and overall gene composition of microchromosomes.

### Sex chromosomes in reptiles

Squamate reptiles epitomize an extremely high level of diversity regarding sex chromosome morphologies, ranging from cryptic (homomorphic), requiring the high-resolution cytogenetic technique of comparative genome hybridization (CGH) to identify them (Ezaz et al. 2005), to highly differentiated sex chromosomes, with these extremes often observed even between closely related species (Ezaz et al. 2009a; Ezaz et al. 2009b; Olmo and Signorino 2005). Yet, only scant genomic information on sex chromosomes and their evolution is available in reptiles. Both male (XY) and female (ZW) heterogametic systems exist in reptiles including multiple sex chromosomes (Ezaz et al. 2009a; Ezaz et al. 2009b; Olmo and Signorino 2005).

Among reptiles, all snakes have ZW sex chromosomes and comparative gene mapping shows that ZW sex chromosomes are conserved across snake lineages (Matsubara et al. 2012; Matsubara et al. 2006). Lizards and turtles, however, have both XY and ZW sex chromosome systems and comparative sex chromosome gene mapping revealed that all lizard and turtle sex chromosomes are non-homologous, implying independent origins of sex chromosomes in these lineages (Ezaz et al. 2009a; Kawagoshi et al. 2012; Kawai et al. 2009; Matsubara et al. 2012; Matsubara et al. 2006; Pokorna et al. 2011).

Compared to whole genome mapping, sex chromosomes (both XY and ZW) of reptiles have been relatively well explored, in particular, comparisons with the chicken Z chromosome. Chromosome painting using the chicken Z chromosome as a probe, gene mapping with a subset of chicken Z chromosome genes, and in silico analysis have been applied to representative species from 22 reptile families (15 lizards, 3 turtles, 3 snakes and 1 crocodilian). These studies revealed non-homology of sex chromosomes between chicken and reptiles (Ezaz et al. 2009a; Pokorna et al. 2011; www.ensembl.org). The chicken Z chromosome is homologous over a substantial region of chromosome 2 in representative species from the majority of the families tested (12/22), suggesting conservation of this chromosome over a long evolutionary time frame (Ezaz et al. 2009a; Pokorna et al. 2011), albeit none of these are sex chromosomes. However, in species from several families of Gekkota (Gekkonidae, Diplodactylidae, Eublepharidae and Pygopodidae), probes for the chicken Z chromosome revealed quite an unusual scenario. For example, five genes from the chicken Z chromosome (*ACO1*/*IREBP*–*RPS6*–*DMRT1*–*CHD1*–*GHR*–*ATP5A1*) are present in the same order on the ZW sex chromosomes of the Hoku gecko, *Gekko hokouensis* (Kawai et al. 2009), implying that either chicken and *G. hokouensis* sex chromosomes have evolved from a common ancestor or such homology is a result of convergent evolution via chromosome rearrangements in this species. However, for species from three other families of Gekkota (Diplodactylidae, Eublepharidae and Pygopodidae) and three other lizard families (Angiudae, Lacerdiae and Teiidae), painting of the chicken Z chromosome showed homology with a medium to small size acrocentric or subtelocentric autosome, not with their sex chromosomes (where present) (Pokorna et al. 2011). Chicken Z chromosome painting also showed homology to chromosome 6 in two turtle species (*Trachemys scripta*, Emydidae and *P. sinensis*, Trionychidae), and a crocodile species tested (*Crocodylus niloticus*, Crocodyldae). It is clear from the above discussion that, although autosomal, the chicken Z chromosome has retained conserved synteny in the majority of reptiles, with the exception of Gekkonidae, where substantial genomic rearrangements may have contributed in an ancestral syntenic break, including coincidental homology between the sex chromosomes of *G. hokouensis* and chicken.

In several recent studies, reptile sex chromosome genes have also been used for cross-species gene mapping. For example, four genes from the Z chromosome of *G. hokouensis* are autosomal in the dragon lizard *P. vitticeps* and five genes from Z chromosomes of snakes and birds are autosomal in the dragon lizard *P. vitticeps* (Ezaz et al. 2009a). Two genes from ZW sex chromosomes of *P. vitticeps* (Ezaz et al. 2013; Young et al. 2013) and five genes from the XY sex chromosomes of marsh black turtle *Siebenrockiella crassicollis* are also autosomal in chicken (Kawagoshi et al. 2012). In addition, an X-linked BAC clone from the green anole is on chicken chromosome 15 (Alfoldi et al. 2011). Such non-homology between avian and reptile sex chromosomes implies multiple and independent origins of sex chromosomes within reptiles, possibly via de novo evolution of sex chromosomes and sex determining genes. However, the de novo evolution can only be verified through targeted studies in those groups with robust phylogeny and evidence of multiple evolution of sex chromosomes within and between species, genera and populations such as in Agamidae and Gekkonidae. A recent in silico comparative study of snake sex chromosomes by Vicoso et al. (2013) presents an excellent example of novel discoveries using genomic and transcriptomic information in a comparative context to understand sex chromosome evolution, degeneration and dosage compensation. This study also provided a platform for comparative analysis of reptilian sex chromosomes, not only with other reptiles but with vertebrates in general.

## Mammalian ancestral karyotype reconstructions

Understanding the events that have led to the diversity observed for mammalian karyotypes requires comparisons to be made across the three major lineages. However, in the study of mammal chromosome evolution, most effort has been placed into determining how the genomes of the karyotypically diverse eutherian mammals have changed since their radiation approximately105 MYA. This is because comparisons with the other two mammalian lineages were impossible using cross-species chromosome painting (Graphodatsky et al. 2012). As a consequence, most studies have focused on reconstructing the eutherian or boreoeutherian ancestral karyotype, rather than on that of the ancestor to all mammals.

Numerous studies have used cross-species chromosome painting to characterize chromosome homology broadly across the eutherian phylogeny as well as in specific eutherian lineages, with these comparisons enabling the reconstruction of the boreoeutherian ancestor (ancestor of most eutherians) or even the ancestral karyotype of all eutherian mammals. Based on cross-species chromosome painting, predominantly with probes to human chromosomes, the genomes of most eutherian species can be divided into 30 to 40 conserved segments (Ferguson-Smith and Trifonov 2007). Exceptions to this level of conservation are observed in species such as dogs and the small apes (gibbons), where the 22 probes to human autosomes detected 73 (Yang et al. 1999) and 51 (Jauch et al. 1992) conserved segments respectively. Overall, chromosome painting with human chromosome probes across the eutherian phylogeny have identified a number of conserved segments corresponding to regions from two or more human chromosomes that are associated in diverse taxa and may therefore, represent an ancestral organization. Several different predicted ancestral eutherian karyotypes have been put forward from these types of analyses, with diploid numbers ranging from 44 to 50 chromosomes (Svartman et al. 2004).

**Fig. 3:**
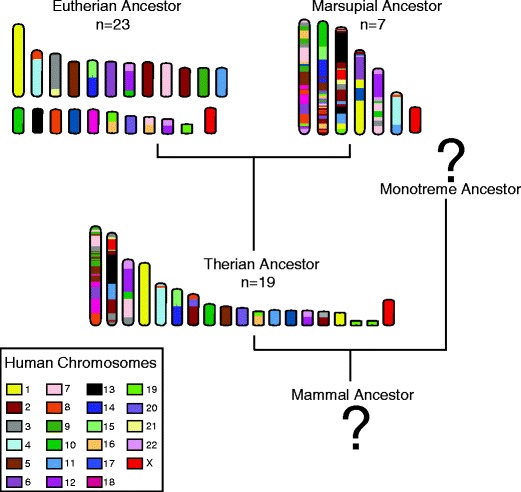
Predicted ancestral karyotypes for mammals, colour-coded for homology to human chromosomes

The availability of well-assembled and anchored genomes for diverse eutherian species has added a greater level of confidence to the predicted ancestral karyotypes, particularly when compared to sequenced outgroup species, such as a marsupial, the South American grey short-tailed opossum (*Monodelphis domestica*), and chicken. Such comparisons have shown that some of the associated segments revealed by chromosome painting amongst eutherian species represent an ancestral organization present in marsupials and even in chicken and the green anole. These associations include the association of segments from human chromosomes 4/8, 12/22, 14/15 and 16/19 (Graphodatsky et al. 2012). The initial attempts made to reconstruct ancestral karyotypes purely from genome sequence comparisons for just a few eutherian species and using chicken as an outgroup, resulted in ‘mammalian’ ancestral karyotypes consisting of 2*n* = 42 (Bourque et al. 2005; International Chicken Genome Sequencing C 2004). The number of adequately assembled and anchored genomes available is one of the major limitations of these types of reconstructions. As more genome sequences have been released, the reconstruction of a putative ancestral eutherian karyotype based on sequence from five eutherian species, opossum and chicken has converged with the widely accepted ancestral eutherian 2*n* = 46 karyotype (Kemkemer et al. 2009), similar to the one depicted in Fig. 3. In-depth comparisons of eutherian ancestral reconstructions have been recently reviewed elsewhere and should be referred to for further insight into how the generally accepted 2n = 46 eutherian karyotype was derived and the supporting evidence (Ferguson-Smith and Trifonov 2007; Graphodatsky et al. 2012; Ruiz-Herrera et al. 2012)

The challenge for more broadly understanding the evolution of mammalian chromosomes now lies with the other two major lineages, the marsupials and monotremes, which diverged from eutherians about 160 (Luo et al. 2011) and 180 MYA (Phillips et al. 2009), respectively. The deep divergence of these three mammalian lineages and the marked differences in their karyotypic features will undoubtedly provide great insight into the events shaping their genomes and the reconstruction of ancestral karyotypes (Deakin et al. 2012b).

### Reconstruction of marsupial ancestral karyotype

Marsupials are renowned for their low diploid chromosome numbers and a high level of conservation across the marsupial phylogeny. The characteristically large chromosomes of marsupials have facilitated cytogenetic studies, with over 70 % of the approximately 330 extant marsupial species karyotyped to date (Hayman 1990). Cross-species chromosome painting using probes derived from the rufous bettong (*Aepyprymnus rufescens*), the marsupial with the highest diploid number of 2*n* = 32, has divided marsupial genomes into just 19 conserved segments (Rens et al. 2003). The speciose Family Dasyuridae show an astonishing level of conservation, as all members of this family karyotyped to date (40 of the 68 known species) have a 2*n* = 14 karyotype (Hayman and Martin 1974a; Rofe and Hayman 1985; Young et al. 1982).

Indeed, marsupial karyotyping studies have reported the predominance of this diploid number across marsupial phylogeny, as well as a 2*n* = 22 chromosome complement. This led to hypotheses for either one of these diploid numbers representing that of the ancestral marsupial (Matthey 1972). These hypotheses were further developed as more cytogenetic information became available. The 2*n* = 14 marsupial ancestor hypothesis is based on this conserved complement being present in six of the seven extant marsupial orders and proposes that fissions of this ancestral karyotype gave rise the higher diploid numbers frequently observed in many marsupial families (Hayman and Martin 1969, 1974a, b; Reig et al. 1977; Rofe and Hayman 1985). The hypothesis for an ancestor with the higher diploid number of 2*n* = 22 proposes that the common 2*n* = 14 karyotype was derived from fusion events early on in the divergence of the different marsupial lineages (Sharman 1973; Svartman and Vianna-Morgante 1998).

Resolving which one of these hypotheses is more likely has been difficult, as evidence to support both hypotheses has been reported over the last 40 years. Support for the 2*n* = 14 hypothesis was substantial, with conservation of the 2*n* = 14 karyotype observed by G-banding (Rofe and Hayman 1985) and cross-species chromosome painting across different families of marsupials (De Leo et al. 1999; Rens et al. 2003; Rens et al. 2001), and a well-resolved phylogenetic tree positioning a 2*n* = 14 species as the basal species (Westerman et al. 2010). Evidence for the alternative hypothesis was weaker. Cross-species chromosome painting demonstrated that, despite the commonality of the 2*n* = 22 diploid number across different marsupial families, the 18 autosomal segments detected were arranged differently between 2n = 22 species (O’Neill et al. 1999; Rens et al. 2003). The main evidence to support the 2*n* = 22 hypothesis was the observation of interstitial telomere signals in members of the Family Didelphidae, a family representing one of the earliest offshoots of the marsupial lineage, with 2*n* = 14 or 2*n* = 18 karyotypes, suggesting that chromosome fusions were responsible for these lower diploid numbers (Carvalho and Mattevi 2000; Svartman and Vianna-Morgante 1998). The inability to compare marsupial chromosome arrangement to outgroup species using chromosome painting prevented further testing of either hypothesis. However, this limitation was recently overcome with the availability of the well assembled and anchored opossum (*M. domestica*) genome (Duke et al. 2007; Mikkelsen et al. 2007) and the sequence and cytogenetic map for the tammar wallaby (*Macropus eugenii*) genome (Deakin et al. 2013; Renfree et al. 2011), enabling comparisons to be made to outgroups such as human and chicken (Deakin et al. 2013; Deakin et al. 2012b).

Comparison of gene arrangement between the two marsupial species and two outgroup species permitted the ancestral marsupial karyotype to be reconstructed, supporting the 2*n* = 14 hypothesis. The arrangement of the conserved segments in the 2*n* = 14 species was observed to be more ancestral than that predicted for the 2*n* = 22 species. The chromosome arrangements of other species could be easily derived, mainly by fissions and inversions, from a 2*n* = 14 karyotype (Deakin et al. 2013).

Although there is strong evidence for a 2*n* = 14 ancestral karyotype, the presence of interstitial telomere signals on *M. domestica* chromosome still needs to be explained. These signals are thought to be the result of a centric fusion but they may actually represent satellite DNA, as C-banding has shown that these signals coincide with pericentric heterochromatin (Pagnozzi et al. 2002). Furthermore, interstitial telomere signals have been observed on marsupial chromosomes that would not have been formed from fusion events from either a 2*n* = 14 or 2*n* = 22 marsupial ancestor. For example, in the 2*n* = 14 fat-tailed dunnart, interstitial telomere signals are observed on chromosome 6 but this chromosome is homologous to an entire chromosome in the 2*n* = 22 common opossum and in a predicted 2*n* = 22 ancestor. This chromosome, however, appears to have undergone at least a couple of large-scale inversions which could have moved telomeric sequences to an interstitial location (Deakin et al. 2012b). Likewise, an inversion event may have occurred in *M. domestica*. Information on gene order for more marsupial species, particularly *Didelphis marsupialis* would help to test this idea.

**Fig. 4:**
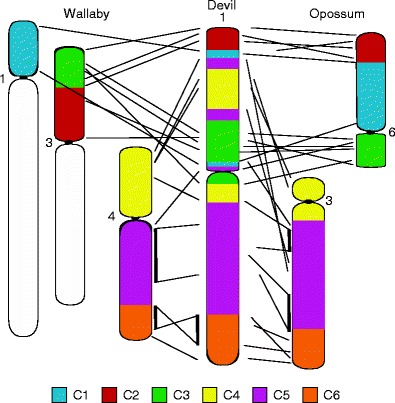
Comparison of gene arrangement between the tammar wallaby, Tasmanian devil and grey short-tailed opossum for conserved segments *C1* to *C6*. Modified from Deakin et al. (2012a, b)

Marsupial chromosomes, when compared by the broad brushstrokes of chromosome painting, may appear to be well conserved but, like bird chromosomes, they have been prone to intrachromosomal rearrangements. A comparison of the opossum genome assembly with the tammar wallaby gene map (Deakin et al. 2013; Deakin et al. 2008), consisting of 554 genes, and the devil gene map consisting of 105 (Deakin et al. 2012a) genes, makes it clear that certain chromosomes have undergone extensive rearrangement since these three species last shared a common ancestor about 80 million years ago. These rearrangements mainly appear to be the result of inversions or a series of inversions, particularly involving conserved segments C1, C2 and C3 (Fig. 4). The 18 conserved segments identified by chromosome painting on marsupial autosomes translated into 76 segments when the tammar wallaby gene map was compared to the opossum genome (Deakin et al. 2013). At this stage, it is unclear how many conserved segments are present when the more lightly mapped devil genome is compared to that of the tammar wallaby or opossum. Mapping of more genes and attempts to improve the genome assembly are currently being carried for more in-depth comparisons.

Analysis of the associations of chromosome segments observed in marsupials and the predicted arrangement in the ancestral marsupial described above, combined with comparisons to the chicken as an outgroup species, has brought us a step closer to deciphering the ancestral karyotype of therian mammals, something that has been difficult to do without the detailed information for more than one marsupial. The predicted ancestral therian karyotype has a 2*n* = 19 chromosome complement (Fig. 3), from which the ancestral marsupial karyotype can be derived by fusion events and the ancestral eutherian karyotype derived from inversions, fissions and fusions (Deakin et al. 2013). This has brought us a step closer to reconstructing the ancestor of all mammals but which ultimately requires information from monotreme genomes (discussed below).

### X chromosomes of therian mammals

Mammals have an XX/XY sex chromosome system. The X chromosome is well conserved in gene content between marsupials and eutherians, with about two thirds of the eutherian X sharing homology with the X of marsupials (Glas et al. 1999). There has been an addition to the X chromosome in the common ancestor of all eutherians (Graves 1995). With the exception of rodents, gene order is remarkably well conserved amongst eutherians, even between humans and the African elephant (Delgado et al. 2009), a representative of the most basal eutherian lineage, the Afrotheria. This is thought to be a consequence of X chromosome inactivation, part of the dosage compensation mechanism to equalize X-borne gene expression between males and females, where rearrangements of the X chromosome may risk disrupting this complex mechanism (Mikkelsen et al. 2007). In contrast, gene order is not conserved between marsupials and eutherians or even between different marsupial species (Deakin et al. 2008). Although marsupials also inactivate one X chromosome in female somatic cells, the mechanism is quite different from that observed in eutherians mammals (reviewed in Deakin 2013) and may be more tolerant of intrachromosomal rearrangements.

### The challenges presented by monotreme genomes

Monotremes, as the earliest offshoot of the mammalian lineage, occupy an important position in amniote phylogeny for the reconstruction of the karyotype for the therian ancestor and the ancestor to all mammals. This lineage consists of just one species of platypus (*Ornithorhynchus anatinus*) and four species of echidna. Monotreme karyotypes feature several large chromosomes and a large number of smaller chromosomes that could not be conclusively distinguished by banding patterns (Wrigley and Graves 1988a, b). It was only when chromosome painting with these small chromosomes was carried out that it became clear that some of them represented different X and Y chromosomes (Rens et al. 2004; Grutzner et al. 2004). Astonishingly, these sex chromosomes form an alternating X and Y chain during male meiosis (Grutzner et al. 2004), and share no gene content with that of the ancestral therian X chromosome, but instead share homology with the Z chromosome of birds (Veyrunes et al. 2008).

Comparisons between platypus and echidna chromosomes have revealed extensive rearrangement between these species. Chromosome painting has shown that platypus chromosomes 1, 4, 5, 9, 11, 14, 16 and 19 correspond to echidna chromosomes 1, 4, 3, 10, 11, 14, 19 and 22, respectively but all other chromosomes are derived, mainly by Robertsonian fissions and fusions (Rens et al. 2007). There are even differences in the composition of the sex chromosomes between platypus and echidna. Male platypuses have five X and five Y chromosomes but the short-beaked echidna (*Tachyglossus aculeatus*) has five X chromosomes and only four Y chromosomes. X_1_, X_2_ and X_3_ are homologous between these two species, however, X_4_ in platypus is homologous to echidna chromosome 27 and echidna X_5_ shares homology with platypus 12p. Reconstructing the ancestral monotreme genome is difficult given that there are so few species in this lineage and the difference between karyotypes (Deakin et al. 2012b; 2013).

To date, monotremes have failed to live up to their potential for aiding in the reconstruction of the therian and mammalian ancestral karyotypes due to several factors. Firstly, as mentioned previously, chromosome paints from other mammalian lineages do not hybridise to monotreme chromosomes (Graphodatsky et al. 2012). Secondly, although the platypus genome has been sequenced, only about a fifth of this sequence has been anchored to chromosomes (Warren et al. 2008), preventing the karyotype of the ancestor to all mammals to be reconstructed. This is largely due to abundant repetitive sequences making it challenging to assemble the genome sequence (Warren et al. 2008). Despite this, the platypus genome assembly has provided some evidence for segmental associations (4q/8q, 12qter/22q, 7a/16p, 3/21, 16q/19q and 22q12/12q24.3) conserved across the Class Mammalia, but this may not be a complete list of all those that are present (Ruiz-Herrera et al. 2012).

Despite the insufficiencies of the platypus genome assembly, as mentioned previously, a therian ancestral karyotype has been predicted (Fig. 3). This prediction obviously has its limitations and having a well-anchored monotreme genome assembly would have provided a greater degree of confidence in the reconstruction (Deakin et al. 2013). Hopefully, continued efforts in monotreme and marsupial genomics will make it possible to test this reconstructed karyotype in the future.

## Conclusions and future directions

Genome technology is making this an exciting time to be exploring the evolution of amniote genomes. However, it is hoped that data from key species in different amniote lineages will help to either confirm current predicted ancestral chromosome arrangements or provide the much needed insight into their reconstruction. For squamate reptiles, it will be appropriate to develop a denser physical map for *P. vitticeps*, a species which already has a preliminary physical map, BAC library and whole genome sequence assembly at a draft stage. This will represent a ZW species, while the green anole genome represents an XY squamate lizard. The tuatara genome is currently being sequenced and the development of a physical map would be appropriate for this lone representative of living Sphenodont. Ideally, at least one species from each major reptilian lineage should have a genome map. Whole genome sequence data from some of the representative groups is already available (e.g. python, king cobra, three species of turtle) so it would be sensible to produce maps for these species. Monotremes are situated at a particularly important phylogenetic location. The echidna genome is also currently being sequenced, yet without being anchored to chromosomes, it will be of little value for studies into genome evolution. It is to be hoped that either a physical map will be produced or that the advances in genome sequencing technology will improve monotreme genome assemblies.

An exciting area of research that needs to be further developed in regards to amniote chromosome evolution is the functional relevance of regions conserved as HSBs and the sequence features contributing to chromosomal rearrangements. For instance, large HSBs conserved across eight eutherians, opossum and chicken are enriched in genes important for development, particularly of the central nervous system and have most likely been conserved as a block to avoid disrupting the important combinations of genes and regulatory elements. In contrast, evolutionary breakpoint regions (EBRs) are commonly enriched for genes related to an organism’s response to external stimuli (Larkin et al. 2009). This also highlights the critical requirement of more anchored amniote genome assemblies for ultimately determining the consequence of chromosomal rearrangements on gene regulation and function that have led to the major phenotypic differences between the different amniote lineages.
